# Investing in the development of the next generation of MCH leaders

**DOI:** 10.3389/fpubh.2025.1606108

**Published:** 2025-07-22

**Authors:** Karen A. McDonnell, Jamal Percy, Lisa Anders, Monique J. Brown, Alice R. Richman, Julianna Deardorff, Monica S. Ruiz, Jihong Liu, Kelli Russell, Audrey Snyder, Cassondra Marshall

**Affiliations:** ^1^Department of Prevention and Community Health, Milken Institute School of Public Health, The George Washington University, Washington, DC, United States; ^2^Kennedy Krieger Institute, Baltimore, MD, United States; ^3^School of Nursing, University of North Carolina—Greensboro, Greensboro, NC, United States; ^4^Arnold School of Public Health, University of South Carolina, Columbia, SC, United States; ^5^Department of Health Education and Promotion, College of Health and Human Performance, East Carolina University, Greenville, NC, United States; ^6^Community Health Sciences, School of Public Health, University of California at Berkeley, Berkeley, CA, United States

**Keywords:** training, maternal and child health, public health, workforce, evaluation

## Abstract

The public health landscape is constantly evolving to address the strengths and needs of the community. Training for the public health workforce is leading the way, establishing an ecosystem approach that integrates individuals within social, political, and environmental contexts to promote health equity within a framework of social justice. One area of public health that is innovatively preparing the next generation of leaders is maternal and child health (MCH). In the United States, key indicators of health disparities within MCH remain stagnant, highlighting the need for training programs that develop future MCH professionals from diverse backgrounds. These professionals will deliver culturally and linguistically appropriate services for an increasingly underserved and underrepresented population, both in the US and around the world. The MCH Leadership, Education, and Advancement in Undergraduate Pathways (LEAP) training program provides coordinated opportunities for undergraduate students, faculty, agencies, organizations, and communities to work together for developing the future MCH public health workforce. Effective and respectful leadership development in MCH requires investment in fundamental educational, research, and community-engaged practice-based skill sets cultivated in undergraduate programs. Currently, six funded programs in the MCH LEAP portfolio share a collective mission to train undergraduates who have historically had a minimal presence to become MCH leaders of tomorrow. These programs also make changes to organizational structures that reflect the geographic and demographic representation of their communities. Mixed-methods evaluations, encompassing both qualitative and quantitative approaches, illustrate the MCH LEAP training program’s effectiveness in introducing and developing the competencies for the next generation of the MCH workforce.

## 1 Introduction

The foundation of an innovative public health ecosystem is a highly qualified, diverse representative workforce equipped with the knowledge and skills to operate across different systems at the individual, family, community, and policy levels. It is also well-documented (1) that groups of individuals with diverse backgrounds and perspectives are more innovative and better at solving complex problems than those that are more homogeneous. Maternal and child health (MCH) in the United States remains one of the most significant and ongoing public health issues confronting society today. Certainly, for the US, one of the wealthiest nations in the world, poor MCH indicators and outcomes should not be the norm. In the US, key indicators for MCH remain stagnant (2), necessitating the creation of an interdisciplinary 21st-century workforce as one of the greatest investment opportunities of our time (3).

It is vital for the MCH workforce to be inclusive to ensure that teams possess the skills to effectively communicate and collaborate in a manner that honors, values, and respects their backgrounds and experiences, and that meets the needs of the communities they serve (4). Improving the health of the nation has always required strong leaders in public health and accountability to their communities. Leaders must critically reassess current needs, programs, and policies for relevance and acceptability, and have the skills to communicate and advocate effectively for solutions (5). Therefore, future MCH public health practitioners must include leaders who possess not only knowledge and skills in MCH and policy but also collaborative, adaptive management, and leadership skills to ensure that new and emerging MCH issues are addressed with ethics, reflection, and continuous learning (6). These developing professionals will provide culturally and linguistically responsive services to increase the involvement among those who have historically been under-supported and had a limited presence, both in the US and globally (7). The MCH Leadership, Education, and Advancement in Undergraduate Pathways (LEAP) training program provides coordinated opportunities for undergraduate students, faculty, agencies, organizations, and communities to work together in developing the future MCH public health workforce and fostering effective, respectful leadership. Leadership development necessitates investment in fundamental educational, research, and practice-based skill sets fostered through undergraduate programs.

The LEAP program demonstrates a commitment to fostering leadership, enriching academic expertise, and promoting diversity within the MCH workforce. The program exposes under-supported undergraduate students from areas that have had few opportunities for health or health-related career paths in the MCH field, including first-generation college students. The program is designed to immerse students in both theoretical aspects, such as open discussions with experts in the field, and practical aspects of MCH, such as applied research and evaluation opportunities. This approach ensures scholars gain a robust academic grounding in MCH and provides them with the tools necessary to address real-world challenges effectively.

Program components include promoting interprofessional collaboration, underscoring the importance of community engagement, and highlighting the need for evidence-based and equity-focused interventions. LEAP ensures emerging health professionals receive essential MCH knowledge and bring cultural humility into their future careers. LEAP also serves as a platform for these scholars to meet others who share similar passions, providing motivation and professional networks that support the students’ continued journey in MCH.

The MCH LEAP program is an investment in training the next generation of professionals to meet emerging public health priorities. Currently, six coordinated programs represent an array of educational programing, with three of the currently funded programs operating within a school of public health and the others in a clinical nursing program, a standalone accredited bachelors in public health program within a college, or as a summer intensive program open to undergraduates from across the US (8). Regardless of the location of the program site or the program structure, the primary goals of MCH LEAP include initiating and implementing the following features:

1 Recruit and support undergraduate trainees to ensure widening involvement through targeted initiatives to:

- Increase the number of undergraduate students exposed to learning opportunities in MCH.
- Promote development and interest in careers in MCH.
- Promote community-tailored approaches and skills in addressing community health needs.

2 Foster development of interdisciplinary leadership and research skills training at the undergraduate level in MCH public health and MCH-related health professions in preparation for careers in MCH.
3 Provide mentorship and internship opportunities to undergraduate students through preceptorships with MCH professionals and faculty, graduate students, and MCH organizations in preparation for graduate education/training in MCH.
4 Increase access to MCH undergraduate education and training through innovative and alternative methods.

The goal of this paper is to share (1) transferable strategies that the LEAP training programs use to recruit, support, and train undergraduate students to be workforce-ready, (2) the process and implementation findings of how the programs foster the development of leaders in preparation for careers in MCH, and (3) formative evaluation findings that incorporate essential lessons learned that could be utilized in other academic or practice settings to build and strengthen academic-practice partnerships.

## 2 Methods

### 2.1 MCH LEAP program descriptions

Each of the six funded MCH LEAP programs is in year 4 out of 5 years of funding. Each has a unique focus on developing undergraduate MCH-related health professionals. The programs are listed below, along with their locations and methods of recruitment, support, and training. Each program has a leadership team that includes a faculty lead as well as supportive faculty and staff. At a minimum, one person at the leadership level dedicates 20% of their time to the MCH LEAP programmatic efforts. Each site determines the structure of the programmatic functions; however, an in-person annual meeting and virtual monthly meetings are held with leadership present for all six funded programs. These monthly meetings serve as brainstorming and roundtable efforts to showcase, strategize, problem-solve, collaborate, and share lessons learned among the sites.

There are three programs located within the programs and schools of public health (George Washington University [GWU], University of California, Berkeley [UCB], University of South Carolina [USC]). These programs provide an institutional pathway for graduate study in public health, in general, and graduate study in MCH, specifically. For two of the sites (ECU and Kennedy Krieger Institute [KKI]), a graduate program in MCH was not available, leading to a partnership with an established MCH MPH program. For those programs located within a school of public health (GW, UCB, and USC), the examination of their undergraduate public health program of study ensures that the course offerings meet the Maternal Child Health Bureau (MCHB) leadership competencies (9).

### 2.2 Process and formative evaluation methods

A formative evaluation was conducted to assess the program process, monitoring and evaluation, and immediate outcomes of the MCH LEAP program. This assessment combined insights from both quantitative (surveys) and qualitative (narratives and interviews) methodologies.

**Figure 1:**
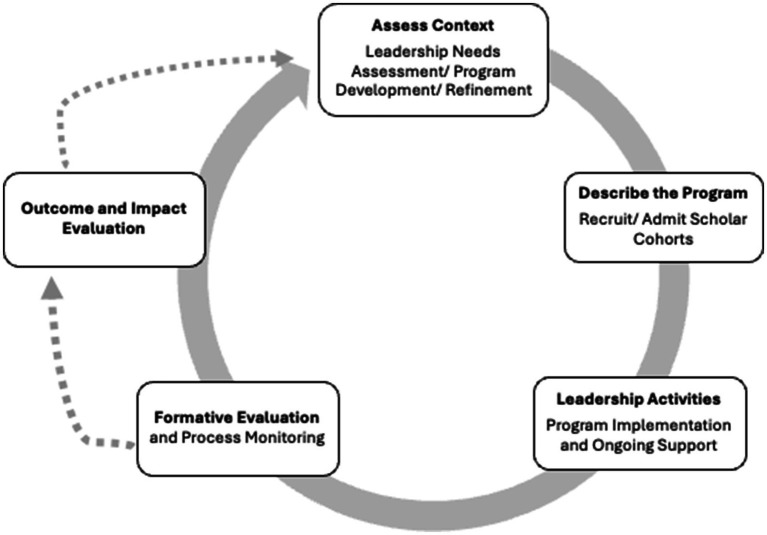
Maternal and child health LEAP evaluation framework.

The overall organization of the MCH LEAP program implementation and evaluation is adapted from the Centers for Disease Control and Prevention (CDC) Program Evaluation Framework and is displayed in Figure 1. The first step in the framework is to assess the programmatic context. For MCH LEAP, this was assessed at both the national and programmatic context level. Each site completed a community needs assessment and an institutional capacity and asset mapping exercise to construct an initial logic model that outlined the institutional resources, activities, anticipated outputs, and outcomes. The resources of the MCH LEAP programs included those at the institutional level, such as the faculty and staff commitment, curricular resources, research and practice opportunities, and scholar recruitment, along with resources at the national level from the Maternal and Child Health Bureau.

The formative evaluation used established domains of success from previously funded MCH workforce training programs (10). Based on the conceptual framework illustrated in Figure 1, these domains included variables that assess recruitment and program activities implementation, including (1) scholar recruitment strategies, (2) scholar demographics, (3) support for scholars, and (4) leadership development and training; these domains were mutually agreed upon by program directors across the various institutions and provided a framework for assessing MCH LEAP’s immediate outcomes and satisfaction; additionally, we expanded our evaluation by including an additional domain; (5) lessons learned, to understand where students felt most satisfied with the program and review various areas of improvement, as this is a relatively new initiative at the program sites.

1. Recruitment strategies: each MCH LEAP site provided the strategies used to recruit cohorts of undergraduate scholars. These activities are listed in Table 1 for each site. In addition, the complete list of strategies was de-identified (institution names were removed), collated, and sorted into thematically similar domains. The resulting themes were then approved by group consensus.
2. LEAP scholar demographics: each MCH LEAP site collected data on race/ethnicity, first-generation college status, and financial need. The sites primarily utilized self-report multiple-choice survey questions completed by the MCH LEAP scholars to evaluate this domain.
3. Supportive mechanisms: each site provided the forms of support introduced at the site to enable scholars to participate in the program activities. Site-specific forms of supportive mechanisms are listed in Table 1. In addition, the complete list of support mechanisms was de-identified, gathered, and sorted into thematically similar domains. The resulting themes were then approved by consensus of group.
4. Leadership development and training: each site provided a schedule of training activities for the LEAP scholar cohorts. Site-specific forms of training activities are listed in Table 1. In addition, the complete list of training activities was de-identified, gathered, and sorted into thematically similar domains. The resulting themes were then approved by consensus of leadership group.
5. Lessons learned: each site implemented data-gathering activities to assess participant satisfaction with program-specific activities and to learn from implementing the new undergraduate MCH leadership programing at their institution. The lessons learned were de-identified by site, and the list was provided to the leadership group. The leadership group engaged in an activity to thematically analyze the resulting themes, which were approved by consensus of group.

**Table 1:** MCH LEAP site strategies to recruit, support, and train workforce-ready students.

LEAP training program	Summarized description
East Carolina University (ECU)	Recruitment: utilizes tailored recruitment flyers, technical assistance videos, outreach via faculty mentors, social media, and presentations in classes. Developed a recruitment video and maintains a dedicated website and LinkedIn profile. Support: provides financial support in the form of stipends and financial support for professional development activities, including conference attendance. Each scholar is paired with a faculty mentor. Training: all scholars complete an MCH Certificate program, a 4-week virtual summer professional development academy, monthly seminars, and an internship. Includes a variety of activities such as guest speakers, conference abstract development, and doula training.
George Washington University* (GW)	Recruitment: dedicated MCH LEAP website, faculty outreach, posted flyer with a quick-response (QR) code leading to the LEAP website, featuring LEAP scholars on recruitment materials. Support: financial support, biweekly in-person cohort meetings, known as “LEAP night,” near-peer, faculty, and community partner mentoring. Training: hands-on training and internships in MCH leadership, research, and practice-based internships. Cohort meetings utilize novel tools outside of academia, such as vision boarding, film, documentaries, book clubs, and museum outings.
Kennedy Krieger Institute (KKI)	Recruitment: sends recruitment information to departments and schools within universities, colleges, and community organizations focused on under-resourced populations. Utilizes website updates, email notifications, and social media. Support: provides a funded 10-week public health leadership training program in the summer. Training: mentored research opportunities and didactic seminars. Scholars present their research at conferences and participate in various events throughout the academic year.
University of California, Berkeley* (UCB)	Recruitment: conducts university-wide outreach using listservs, newsletters, social media, classroom presentations, student fairs, and info sessions. Enables active LEAP scholars to promote the program. Support: provides support with the public health primary application process, peer advising, and graduate student and faculty mentorship. Utilizes cascading mentorship pods and offers a three-semester seminar for community-building and skills development. Training: offers a 1.5-year MCH undergraduate curriculum, including coursework, internships, research fellowships, and leadership training. Scholars attend a Summer Bridge Course and a leadership retreat.
School of Nursing, University of North Carolina – Greensboro (UNCG)	Recruitment: outreach to students enrolled in prelicensure BSN and RN-to-BSN nursing programs, outreach to local high schools. Support: professional development mentoring, career, and graduate school application guidance, MCH LEAP scholar dedicated tutor, and National Council Licensure Examination (NCLEX) preparation materials, financial support for professional MCH organization membership. Training: monthly seminars, supplementary materials for existing nursing courses, MCH-related simulation training and certification course offerings (e.g., doula training and neonatal resuscitation), shadowing, and MCH placement opportunities for clinical rotations.
University of South Carolina* (USC)	Recruitment: uses various strategies, including flyers, social media, Zoom meetings, and student fairs. Developed a recruitment video with student testimonials. Support: pairs trainees with faculty mentors, provides ongoing mentorship, and assists with internship applications and professional development. Training: offers monthly dinner seminars, an MCH Spring Summit, and presentations of research projects. Provides career-development learning and networking opportunities.

*School/Program of Public Health.

## 3 Results

A primary objective of MCH LEAP is to recruit and support undergraduate trainees to ensure widening exposure to leadership and learning opportunities in MCH. As displayed in Table 1, all MCH LEAP programs have recruitment, support, and training program components tailored to their academic location.

### 3.1 Recruitment

All the programs engage in active recruitment that leverages existing undergraduate programing efforts. Thematic analysis of the site’s recruitment strategies yielded six domains, including targeted outreach, the use of digital and multimedia tools, engagement with current scholars, informational sessions and presentations, and inclusive and intersectional approaches.

Each program has an annual formal application process that ensures participants are knowledgeable about the requirements of participation and that the cohort size selection matches resource availability. The MCH LEAP program has recruited four cohorts, with each site cohort including 10–32 scholars. All MCH LEAP scholars represent demographic backgrounds that have had limited representation in MCH leadership, training, and the workforce.

### 3.2 MCH LEAP scholar demographics

The site-specific and total sample demographics of the four cohorts are reported in Table 2. A total of 325 undergraduates have participated in MCH LEAP across the six programs in 4 years, with 95% self-identifying as female and half as African–American/Black and nearly one-fifth (18%) Hispanic/Latine, for those programs that systematically collected additional demographic data, a majority qualified as financially in need (Pell Grant/Stafford/Work–Study (WS) eligible), half identified as first-generation college students, and over two-thirds were employed in a part time or full time position while pursuing their undergraduate degree.

**Table 2:** MCH LEAP self-reported demographic information.

	ECU	GW	KKI	UCB	UNCG	USC	Total
	*N* = 61	*N* = 43	*N* = 39	*N* = 34	*N* = 30	*N* = 118	*N* = 325
Gender identification							
Female	56 (92%)	42 (98%)	34 (87%)	31 (91%)	29 (97%)	116 (98%)	308 (95%)
Male	4 (6%)	1 (2%)	5 (13%)	1 (3%)	1 (3%)	2 (2%)	14 (4%)
Non-binary	1 (2%)			2 (6%)			3 (1%)
Race							
African–American/Black	29 (48%)	21 (49%)	19 (49%)	1 (3%)	14 (47%)	77 (65%)	161 (50%)
American–Indians/Alaska Natives		1 (2%)		4 (12%)			5 (2%)
Asian		7 (16%)	8 (51%)	16 (47%)	4 (13%)	6 (5%)	31 (10%)
Multiple	6 (9%)	2 (5%)	3 (8%)				11 (3%)
White	19 (32%)	12 (28%)	6 (15%)	7 (21%)	9 (30%)	18 (15%)	71 (22%)
Not answered	7 (11%)		3 (8%)	6 (18%)	3 (10%)	17 (14%)	36 (11%)
Ethnicity							
Hispanic/Latine	7 (11%)	11 (25%)	8 (21%)	18 (53%)	6 (20%)	9 (8%)	57 (18%)
Financial aid (Pell/Stafford/WS)							
Yes		33 (77%)		18 (53%)		74 (63%)	
First generation							
Yes	30 (50%)	17 (40%)	13 (33%)	24 (71%)			
Employment status							
Full time	4 + (11%)	1 (2%)		2 (6%)			
Part time		29 (67%)		28 (82%)			
Not currently employed		13 (30%)		4 (12%)			

### 3.3 Support for scholars

MCH LEAP programs have developed a cohort-based interactive training program that includes regular periodic meetings with LEAP faculty and staff. Support for LEAP scholars takes various forms at each site. Responses from LEAP scholars have noted that MCH LEAP programs support scholars by holding regular cohort meetings, providing a psychologically safe space for scholars to ask questions, learn from one another, share experiences without fear of needing to “code-switch,” and instill a sense of belonging. A key area of support for the scholars is the provision of financial resources, including stipends and sponsorships, to attend professional conferences or professional development opportunities. The provision of financial resources is instrumental in providing scholars, many of whom, as listed in Table 2, are facing significant financial constraints and competing demands, and without this support, would be unable to participate in the program.

A fundamental aspect of the MCH LEAP program is the provision of diverse mentoring at multiple levels. Mentoring is an intentional and purposeful process, involving faculty, MCH professionals, and peers. Faculty, staff, and graduate students serve as mentors, representing various roles in leadership, professional development, and research. MCH professionals mentor scholars by sharing their expertise in community MCH efforts. An area of mentorship that has flourished as cohorts complete the training program is near-peer mentoring, where MCH LEAP scholars from one year mentor subsequent cohorts. Mentoring can be either individual or team-based, addressing leadership, research, and practice in workforce development. Regardless of the type of mentorship, central to effective mentoring is the creation of contracts that establish roles, responsibilities, and expectations between mentors and scholars as mentees.

### 3.4 Leadership development and training

**Figure 2:**
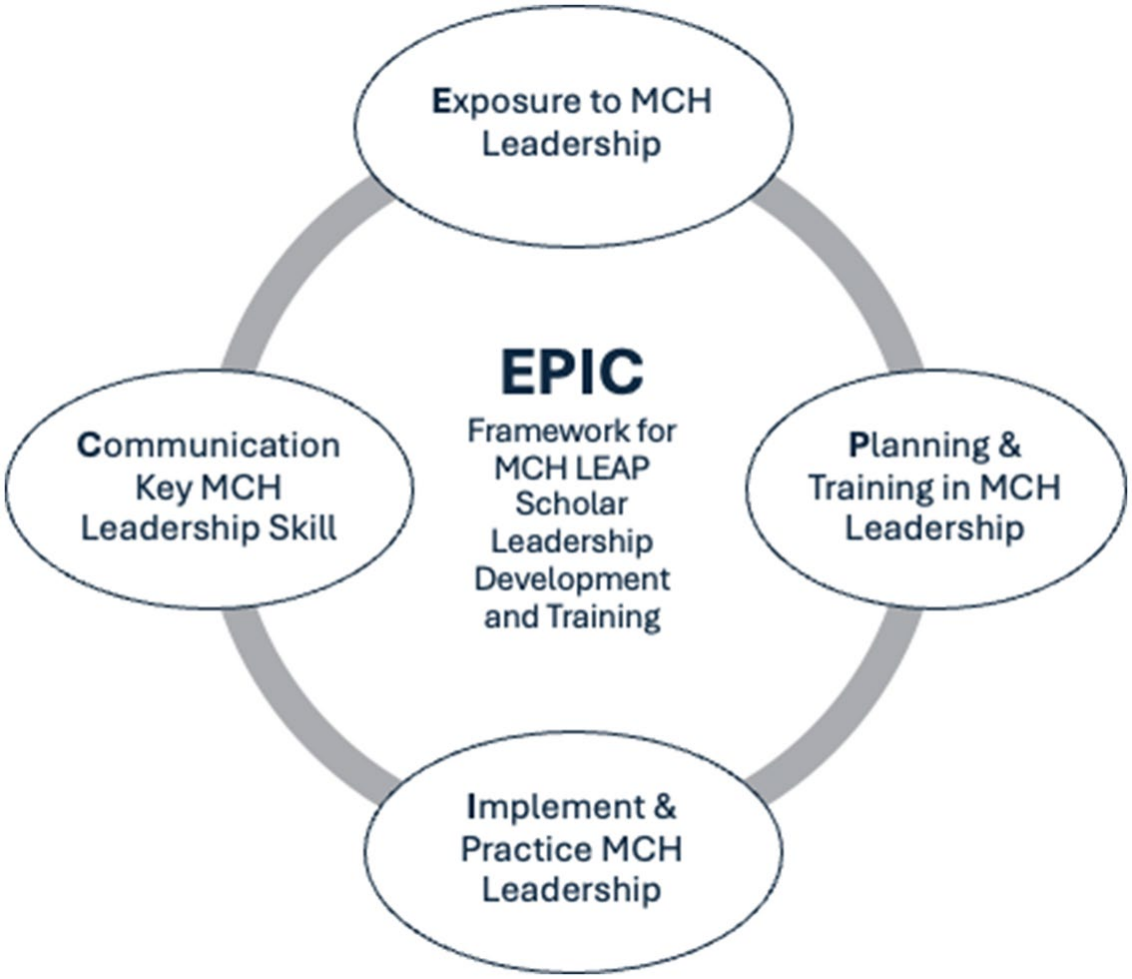
Maternal and child health LEAP EPIC framework.

The training programs have a site-specific structure and function that meets the capacities and opportunities of the institution. The choice of leadership activities is based on the MCH LEAP-developed Exposure, Planning, Implementation, and Communication (EPIC)Framework displayed in Figure 2, and specific activities are listed in Table 3. As part of the preliminary activities of LEAP, there is exposure to MCH Leadership through structured academic programing, MCH professional panels, discussions, as well as creative endeavors, including vision boarding activities. After exposure, scholars engage in a structured Individual Leadership and Development Planning (ILDP) process whereby the scholars, with guidance from the program, plan the MCH leadership training activities. The planning phase evolves into the implementation and practice phase, where scholars engage in hands-on training, research, and practice internships, as well as leadership skill development in MCH. A key skill set developed within the MCH LEAP training activities is effective communication. MCH LEAP scholars utilize supportive training activities, including institutional career centers, formal professional development institutes, and academic programing. MCH LEAP scholars practice their skill sets in communication by engaging in presentations of their leadership activities in a variety of channels and formats, including oral, written, in-person, blog posts, website posts, academic poster presentations, and manuscripts.

**Table 3:** MCH LEAP undergraduate leadership training activities.

Theme	Training program activity example
Hands-on training and internships	Internships and practicums with various contacts, such as practitioners and faculty mentors A 160-h summer internship and a 150-h research fellowship with MCH community partners Summer internships application support Experiential learning opportunities
Structured academic programs	MCH Certificate program with foundational courses, MCH undergraduate curriculum, including coursework, internships, and research fellowships
Professional development	A 4-week virtual summer academy with guest speakers, conference abstract development, and professional preparations 10-week public health leadership training program with mentored research opportunities and didactic seminars Monthly dinner seminars and an MCH Spring Summit with career roundtables and networking opportunities
Leadership skill development	Resumes, cover letters, interview skills, and personal branding development Leadership retreat and participation in leadership training throughout the program Guidance on the graduate school application process
Seminars and workshops	Monthly seminars on various MCH-related topics, including team building, research presentations, and professionalism Didactic seminars on public health topics with an emphasis on health disparities and social determinants Monthly dinner seminars with guest speakers and career-development learning
Community and peer learning	Peer review to strengthen poster submissions to conferences Cascading mentorship pods and a three-semester seminar for community-building and skills development Presentations of research projects by “near-peer” students and graduate students

### 3.5 Lessons learned

Collective responses from the programmatic leadership and qualitative responses from the scholars yielded eight common themes in lessons learned from the six LEAP sites regarding formalized MCH leadership development. The first theme centered on presence and availability. This theme emphasized the importance of programmatic faculty and staff being present and available for the scholars through regular contact and informal support, highlighting the value of in-person meetings and non-academic engagement to build relationships. The second theme focused on strong mentorship and team building, emphasizing a dedicated project team with diverse multimember mentorship, and a mutually agreed-upon contract between mentor and mentee, ensuring a formalized process.

A central theme involves instituting evaluation and feedback mechanisms. Each site reported the importance of implementing systematic forms of evaluation and continuous quality improvement from the program’s inception. The importance of ensuring financial support to the scholars was a key theme underscoring the necessity of stipends and support for professional development, training, and educational expenses. This theme is closely tied to the concept of understanding and addressing student needs. As undergraduates, the MCH LEAP sites recognize the unique psychosocial stressors faced by underrepresented scholars and the balance they must maintain between academic, work, and personal responsibilities, while being understanding and supportive. This theme is intertwined with the sixth theme of utilizing technology and systems, not only in creating MCH leaders, but in communicating with scholars and recognizing the potential for technology to shape the MCH workforce today and in the future. The seventh theme for lessons learned focused on community and relationship building. Within this theme, we are taking steps to celebrate the collaborative academic/community programmatic wins collectively and to be sure to highlight community partnerships as they play an integral role in engaging and training the scholars. Finally, the importance of creating MCH leaders of tomorrow who can engage professionally with each other and with the broader MCH community. These themes reflect the collective experiences and insights from LEAP sites, providing valuable lessons for recruiting, supporting, and training undergraduate students in MCH.

## 4 Discussion

The MCH LEAP program is designed to prepare undergraduate students for careers in maternal and child health (MCH) leadership through hands-on experiential learning, cohort-based professional development, leadership training, and mentorship opportunities. The program’s objectives are achieved using the MCH LEAP EPIC framework, which structures site-specific approaches to train a diverse group of students. Undergraduates are recruited into a cohort of scholars, providing them with exposure to various career paths, supportive mentorship, and team-building activities. This foundational approach leverages individual learning and development plans to guide training in MCH leadership. Activities include professional development, skill-building, and experiential learning through research, practice, community-based internships, and other professional opportunities. A key aspect of the MCH LEAP scholar training program is to enhance communication skills across various media and formats, essential for effective leadership in MCH.

The MCH LEAP program in these sites has completed four cohorts of recruitment and training of undergraduate scholars. The recruitment strategies vary from informational sessions/presentations to targeted outreach to the use of digital/social/multimedia tools and have demonstrated success in meeting the LEAP objective to recruit and support undergraduate trainees to ensure widening involvement through targeted initiatives to increase the number of undergraduate students exposed to learning opportunities in MCH. The six LEAP programs have recruited over 300 scholars from backgrounds that have been under-supported and had limited opportunities for engagement in MCH leadership. Although each program designs supportive training mechanisms that fit its capabilities, the leadership skills being developed are noteworthy. Taken together, the MCH LEAP program posits that there are six key strategies that each program must integrate for practical MCH leadership training for undergraduates in preparation for ensuring the future MCH workforce mirrors the population (see Table 4).

**Table 4:** Six key strategies for MCH undergraduate leadership training programs.

Have a mechanism for community and relationship building, such as a cohort model.
Establish a formalized mentorship program.
Implement professional development and skill-building strategies.
Provide exposure to different career paths.
Ensure students have financial support.
Integrate methods of evaluation and continuous improvement

The first strategy is that the program must have a mechanism for building community and relationships. The use of a cohort model for recruitment was intentional as a means of creating a sense of belonging among the LEAP undergraduate scholars. Holding regular (weekly, biweekly, or monthly) in-person meetings helps to cultivate a sense of belonging and relationship building through non-academic engagement. The incorporation of a cohort model provides the space for the second strategy, having a formalized mentorship program. The mentoring teams should include members of the MCH faculty, MCH organizations, and the greater MCH community. As cohorts progress, near-peer mentoring offers several benefits for both mentors and mentees, including increased access and communication. Near-peer mentors often provide more frequent and accessible communication compared to traditional mentors, allowing for stronger relationships and more personalized guidance (11–13). Since near-peer mentors have recently navigated similar experiences, they can offer relevant advice with a level of empathy and understanding that is particularly valuable. Near-peer mentors often report increased confidence and a stronger sense of belonging as a result of participating in LEAP activities, which can enhance their own professional and personal development and understanding of self-efficacy. On the academic front, studies have shown that near-peer mentoring can positively impact mentees’ persistence in their educational and career paths, particularly in fields like MCH (14, 15). Finally, there is an opportunity for mutual learning and growth as both mentors and mentees benefit from the exchange of knowledge and experiences, fostering a collaborative learning environment.

A third strategy is for each program to have professional development and skill-building strategies in place. These programs may include structured multi-week professional development courses, research internships, or practice-based opportunities. The key aspects of these strategies are to ensure there are opportunities for professional workforce exposure and readiness activities for the scholar, including research seminars, professional shadowing, and networking opportunities. These opportunities may already be available in the institutions’ undergraduate programs, and their effective use should be leveraged to create MCH LEAP-specific meetings and resources to guide the cohort, as many are first-generation college students.

A fourth strategy is exposure to different career paths through the MCH leadership training program, such as pursuing a pathway in research, clinical care, community programing, or policy analysis. The MCH workforce encompasses a variety of pathways, and exposure to this diversity needs to be intentional. Activities such as panels featuring MCH professionals and alumni, visits to graduate programs, shadowing opportunities, internships, and encouragement to collaborate with the local MCH community of professionals are some of the mechanisms that have been implemented. To take full advantage of these opportunities is the providence of the fifth strategy of a successful undergraduate pathways training program, financial support. As demonstrated in the students involved in the four MCH LEAP cohorts, the majority qualify for financial need, and the provision of stipends and support for participation in the MCH LEAP training program is vital. Without such dedicated financial support, students would not be able to forgo paid opportunities in non-public health/MCH sectors to take advantage of these and other professional opportunities, which may or may not be paid, thus may further set back their readiness for graduate school and participation in the MCH workforce.

Finally, a key strategy for establishing similar training programs is the use of techniques for evaluation and continuous improvement. These need to be multifaceted and ready for implementation prior to the program’s rollout. For MCH LEAP, each site assessed the programmatic context that included a community needs and institutional capacity assessment. This effort was instrumental in determining the institutional resources and activities required to achieve the program’s goals and objectives. Evaluation includes program process, monitoring, and evaluation, as well as immediate outcomes for the MCH LEAP program. The program evaluation plan should combine multiple methods to form a mixed-methods design, utilizing both quantitative and qualitative methodologies. Taken together, these leadership strategies are designed to foster the development of diverse leaders in maternal and child health, ensuring they are well-prepared for their future careers.

### 4.1 Strengths and limitations

The MCH LEAP program and associated recruitment, support, and training strategies have been successfully integrated into the six sites represented. The information presented details the development of the institutionally based programs and the processes that were implemented. As three of the programs are based within schools of public health, the transferable strategies may face facilitators or challenges that were not present for these sites if implemented outside of a school of public health. However, one site was based in a clinical program, one in an accredited public health program within a college department, and one as a standalone summer internship program, thereby enhancing the diversity of environments involved in training MCH LEAP scholar cohorts. A limitation of this evaluation is that it is formative, with findings grounded in a predominantly qualitative approach utilizing thematic analyses based on a consensus approach. Future evaluations of the MCH LEAP program should incorporate an outcome evaluation that includes the MCH LEAP scholars, with a focus on both immediate and long-term MCH leadership and work force.

## 5 Conclusion

The MCH LEAP training program builds a pathway for undergraduate students to become workforce-ready through cohort-based professional development, hands-on experiential learning, and mentorship opportunities. Each MCH LEAP training program employs a site-specific, tailored approach to recruit, support, and train undergraduate students in preparation for careers in MCH. The MCH LEAP EPIC model guides the selection of activities to provide exposure to diverse careers in MCH, as well as training in research, practice, and communication skill sets necessary for future MCH leaders. These academic practice partnerships, such as MCH LEAP, are mutually beneficial and promote knowledge sharing, enhance lifelong learning, and support the long-term success of the MCH workforce, which will directly improve the public’s health.

## Data Availability

The original contributions presented in the study are included in the article/supplementary material, further inquiries can be directed to the corresponding author.
